# Comparative Study on Serum Levels of 10 Trace Elements in Schizophrenia

**DOI:** 10.1371/journal.pone.0133622

**Published:** 2015-07-17

**Authors:** Tiebing Liu, Qing-Bin Lu, Lailai Yan, Jing Guo, Fangbo Feng, Jinyun Qiu, Jingyu Wang

**Affiliations:** 1 School of Public Health, Peking University, Beijing, People’s Republic of China; 2 Civil Aviation Medicine Center, Civil Aviation Administration of China, Beijing, People’s Republic of China; 3 Department of Clinical Laboratory, PLA 261st Hospital, Beijing, People’s Republic of China; The Florey Institute of Neuroscience and Mental Health, AUSTRALIA

## Abstract

The etiology and pathophysiology of schizophrenia remain obscure. This study explored the associations between schizophrenia risk and serum levels of 10 trace elements. A 1:1 matched case-control study was conducted and matched by age and sex. Blood samples were collected to determine the concentrations of nickel, molybdenum, arsenic, aluminum, chromium, manganese, selenium, copper, iron and zinc by an inductively coupled plasma-mass spectrometry. The conditional logistic regression model was used to analyze the associations between trace elements and schizophrenia risk. Totally 114 schizophrenia patients and 114 healthy controls were recruited in the study. The multivariate analysis demonstrated that copper≤0.97 μg/mL, selenium≤72 ng/mL and manganese>3.95 ng/mL were associated with an increased risk of schizophrenia. The study showed that lower levels of selenium, copper and higher levels of manganese were found in schizophrenia patients compared with healthy controls.

## Introduction

Schizophrenia is a major mental illness characterized by symptoms including psychosis, apathy and social withdrawal, and cognitive impairment [1]. In 2010, schizophrenia caused 13.6 million disability-adjusted life years (DALYs), accounting for 7.4% of DALYs caused by mental and substance use disorders [2]. A systematic review has reported the median lifetime prevalence of schizophrenia was 4.0 per 1000 persons in the world [3], and 4.9 per 1000 persons in China [4]. In spite of decades of researches, the etiology and pathophysiology of schizophrenia remain unclear. However, schizophrenia has been almost considered as a multifactorial disorder with genetic and environmental factors contributing to overall risks [5].

Environmental risk factors associated with schizophrenia risk mainly include cannabis use, prenatal infection or malnutrition, perinatal complications, and a history of winter birth, etc. [6, 7]. However, the mechanisms of these risk factors are still unknown.

Trace elements play a versatile function in the biological system ranging from regulating metabolic reactions to acting as antioxidants [8, 9]. Numerous studies have suggested that alterations of trace element levels may be associated with the etiology and pathophysiology of several psychiatry disorders [8, 10–13], including schizophrenia [14–17]. A prospective study has indicated that maternal iron deficiency may be a risk factor for schizophrenia among offspring [18].

However, there have been considerable professional debates on the associations between some trace elements and the risk of schizophrenia, e.g. copper. The decreased, increased and unchanged copper levels in patients with schizophrenia have been reported by different studies [15, 19–22]. In addition, some other trace elements have not been involved or fully discussed, such as nickel, manganese and aluminum.

Therefore, a matched case-control study was performed to explore the associations between serum concentrations of 10 trace elements and the risk of schizophrenia in China.

## Materials and Methods

### Study subjects

The 1:1 matched case-control study was conducted from November 2012 to April 2013 in the 261 Hospital of People's Liberation Army, Beijing, China. The cases were the patients with the ICD-10 diagnosis of schizophrenia (F20) recruited from consecutive admissions at the psychiatric department. Patients who suffered from schizophrenia together with some other psychiatric disorders were also excluded from the study. The controls were individuals without any known psychiatric problem matched with cases by the similar age and sex. All subjects had to be ensured that they did not have diabetes, kidney failure, or other diseases, nor had they been treated with mineral or vitamin supplements that might alter levels of trace elements. In the study period, 114 cases and 114 controls fulfilled the inclusion criteria. The study protocol was reviewed and approved by the Ethics Review Committee of Health Science Center, Peking University (IRB00001052-12065). The study subjects were informed about the purpose of the study, and written consent was obtained from each of them. If the independent capacity of any study participant was doubted, the written consent of the relevant primary caregiver was obtained simultaneously.

### Sample collection and storage

About 5 mL venous blood was drawn from the forearm vein of each subject after an overnight fast, using a plastic syringe with a stainless steel needle. Each blood sample was then collected into a metal-free plastic tube, and clot at room temperature for half an hour and then centrifuged at 3,000 rpm for 15 minutes. Serum samples were all stored at −20°C and also protected from light. In order to prevent metal contamination, all tubes used were made of polypropylene instead of glass materials. Blood collection and separation were both carried out in a dust-free room.

### Instrumentation and trace element determination

Each serum sample was put into a quartz tube and 1.5 mL purified HNO_3_ (nitric acid) was added. After predigestion at room temperature for two hours, 0.5 mL H_2_O_2_ was added to promote further digestion. The tubes were then placed in a microwave digestion system (MWS-2; Bergholt Co., Germany) and diluted to 7 mL with deionized water. Concentrations of nickel, molybdenum, arsenic, aluminum, chromium, manganese, selenium, copper, iron and zinc were determined by an inductively coupled plasma-mass spectrometry (ICP-MS, American PerkinElmer ELAN DRCII).

### Data analysis

Statistical analyses were performed using SAS 9.1.3 (SAS Institute, Cary, North Carolina). For association analyses, participants were divided into three groups based on the normal values in the serum for iron, copper, selenium, zinc, arsenic, aluminum, chromium, nickel, manganese and molybdenum and the distribution of trace element concentrations among controls [23–25]. A conditional logistic regression model was used to analyze the associations between serum trace elements and the risk of schizophrenia. The variables significantly associated with the risk of schizophrenia (*P*<0.05) in univariate analysis were selected into the multivariate conditional logistic regression model. Odds ratios (*ORs*) and 95% confidence intervals (95% *CIs*) were calculated. A two-sided *P* value of less than 0.05 was considered to be statistically significant.

## Results

Totally 114 schizophrenic patients as cases and 114 health volunteers as controls were recruited in the study. There were 76 pair-males and 38 pair-females. The mean ± standard deviation (SD) of age were 32.8±11.3 years and 33.0±10.7 years in the case and control groups, respectively (*P* = 0.683).

Ten trace elements were analyzed in the study, including iron, copper, selenium, zinc, arsenic, aluminum, chromium, nickel, manganese and molybdenum. The univariate analysis by conditional logistic regression models demonstrated that the levels of eight trace elements were associated with the risk of schizophrenic (*P*<0.05) except zinc and molybdenum (Table 1). Specifically, lower concentrations of iron, copper, selenium, arsenic and aluminum, as well as higher concentrations of chromium and manganese were associated with an increased risk of schizophrenia. Interestingly, the lower nickel concentration revealed a significant association with the risk of schizophrenia.

**Table 1:** Distribution of the trace elements in the case and control groups and univariate analysis for the associations between trace elements and schizophrenia by conditional logistic regression models.

Trace Element	Cases	Controls	OR (95%CI)	P-value
Iron, μg/mL				
≤0.86	21	7	3.044 (1.102–8.408)	0.032
0.86~1.87	82	83	1.000	
>1.87	11	24	0.457 (0.186–1.119)	0.086
Copper, μg/mL				
≤0.97	92	60	5.166 (2.297–11.618)	<0.001
0.97~1.64	21	47	1.000	
>1.64	1	7	0.255 (0.029–2.216)	0.215
Selenium, ng/mL				
≤72	86	13	40.768 (9.557–173.908)	<0.001
72~224	27	98	1.000	
>224	1	3	3.368 (0.131–86.380)	0.463
Zinc, μg/mL				
≤0.67	19	23	0.794 (0.418–1.509)	0.482
0.67~1.83	93	87	1.000	
>1.83	2	4	0.481 (0.088–2.638)	0.399
Arsenic, ng/mL				
≤20	46	6	12.434 (3.827–40.395)	<0.001
20~43	59	80	1.000	
>43	9	28	0.464 (0.201–1.068)	0.071
Aluminum, μg/mL				
≤0.11	46	82	1.000	
0.11~0.78	66	30	3.790 (2.054–6.995)	<0.001
>0.78	2	2	2.766 (0.341–22.419)	0.341
Chromium, ng/mL				
≤0.20	3	2	1.953 (0.314–12.131)	0.473
0.20~2.00	49	66	1.000	
>2.00	62	46	1.891 (1.075–3.326)	0.027
Nickel, ng/mL				
≤2.6	41	0	-*	<0.001
2.6~7.8	54	59	1.000	
>7.8	19	55	0.350 (0.148–0.828)	0.017
Manganese, ng/mL				
≤2.36	6	38	0.837 (0.255–2.752)	0.770
2.36~3.95	8	39	1.000	
>3.95	100	37	29.882 (6.628–134.722)	<0.001
Molybdenum, ng/mL				
≤1.59	57	38	1.754 (0.933–3.296)	0.081
1.59~2.07	32	39	1.000	
>2.07	25	37	0.790 (0.375–1.662)	0.534

*means that the OR and 95%CI cannot be calculated as one cell was zero.

When we merged the groups with a small sample size, the variables with *P*<0.05 in the univariate analysis except nickel were entered into a multivariate conditional logistic regression model (Table 2). The results showed that copper≤0.97 μg/mL, selenium≤72 ng/mL and manganese>3.95 ng/mL were associated with an increased risk of schizophrenia, for which the ORs were 20.957 (95%CI: 1.381–318.141), 16.837 (95%CI: 2.130–133.113) and 19.269 (95%CI: 1.436–258.626), respectively.

**Table 2:** Multivariate analysis for the associations between the trace elements and schizophrenia by conditional logistic regression model.

Trace Element	OR (95%CI)	P-value
Iron, μg/mL		
≤0.86	5.802 (0.869–38.752)	0.070
>0.86	1.000	
Copper, μg/mL		
≤0.97	20.957 (1.381–318.141)	0.028
>0.97	1.000	
Selenium, ng/mL		
≤72	16.837 (2.130–133.113)	0.007
>72	1.000	
Arsenic, ng/mL		
≤20	12.658 (0.761–210.627)	0.077
>20	1.000	
Aluminum, μg/mL		
≤0.11	1.000	0.355
>0.11	2.434 (0.369–16.034)	
Chromium, ng/mL		
≤2.00	1.000	
>2.00	7.106 (0.855–59.071)	0.070
Manganese, ng/mL		
≤3.95	1.000	
>3.95	19.269 (1.436–258.626)	0.026

## Discussion

The present study explored the associations between serum levels of 10 trace elements and the risk of schizophrenia, demonstrating that lower concentrations of copper, selenium and nickel and higher concentrations of manganese were associated with an increased risk of schizophrenia. However, the association between iron deficiency and schizophrenia risk was not significant after adjusted for other trace elements, including copper, selenium, arsenic, aluminum, chromium and manganese.

Epidemiological evidence regarding the association between serum copper levels and schizophrenia risk is controversial. Several studies in the United States and Serbia reported increased plasma copper in schizophrenia patients compared to healthy controls [19, 20], whereas one study in Romania observed an insignificant decrease among cases. These inconsistent findings might result from racial factors, and relatively small sample sizes of previous studies. In this study, we found that lower levels of copper were associated with an increased risk of schizophrenia, which was similar to the results reported by a previous study [21]. The alteration of copper level has been relevant in the etiology or pathogenesis of several mental diseases [8, 26], including schizophrenia [15, 22]. Copper plays an essential role for the activities of several physiologically enzymes including cytochrome c oxidase and superoxide dismutase, which are important for diminishing oxidative damage [26]. Moreover, copper-containing enzymes, including dopamine-beta-hydroxylase and tyrosine hydroxylase, are linked to the synthesis of dopamine and norepinephrine, which have been involved in the etiology of schizophrenia [22]. Therefore malfunctions related to any of these enzymes due to lack of copper might be associated with the pathogenesis and etiology of schizophrenia.

Selenium plays a key role in the function of the glutathione peroxidase antioxidant system, which can convert hydroxyl radicals and peroxides into nontoxic forms [27]. Therefore lower activity of glutathione peroxidase (GPx) induced by selenium deficiency might contribute to oxidative stress which has been linked to schizophrenia. This was also supported by Vural et al., who noted that erythrocyte GPx activity and plasma selenium concentration were significantly lower in patients with psychiatric disorders [26]. Several studies also observed that high levels of selenium in peripheral blood were inversely associated with several psychiatric disorders, including schizophrenia[28, 29]. Similarly, the selenium concentration was lower in schizophrenia patients compared with controls in this study.

Excess exposure to manganese has been known to induce neurotoxicity and high concentrations of this trace element were reported to accumulate in the mitochondria of brain areas [30]. In animal study, manganese toxicity has been involved in DNA fragmentation[31] which was observed in schizophrenia [32]. In postmortem frontal cortex of individuals with schizophrenia, altered levels of extracellular signal-regulated kinase (ERK) signaling proteins were observed, whose activations were linked to Mn-induced neurotoxicity via mitochondrial-dependent pathways [33, 34]. In addition, several studies regarding the relationship between alterations of blood (serum or plasma) or hair manganese levels and psychiatry disorders suggested that manganese might play a role in the pathogenesis of psychiatry disorders via either its nutritional or toxicological properties [10, 12, 13, 30]. Similarly, higher manganese levels were associated with an increased risk of schizophrenia in the present study. However, one study did not find an alteration of manganese hair levels between schizophrenia patients and controls in a small sample size (30 cases *vs*. 30 controls) [15].

Iron is essential for the synthesis of hemoglobin which is responsible for blood oxygenation. A prospective study on an association between maternal iron deficiency and the risk of schizophrenia in offspring indicated that a 27% decrease in the rate of schizophrenia spectrum disorders for every 1 g/dL increase in mean maternal hemoglobin [18]. Iron also plays an important role in catalytic activity of catalase (CAT), which is essential for the antioxidant system [26]. Raffa et al. found that CAT activity was significantly lower in schizophrenia patients, suggesting a link between iron deficiency and schizophrenia [35]. In addition, Iron is a coenzyme of dopamine synthesis; therefore iron deficiency might influence the density and activity of dopamine receptors, which in turn has been implicated in the pathogenesis of schizophrenia. In the present study, we observed an association between lower levels of iron and an increased risk of schizophrenia according to the univariate analysis; however, the association was not significant when adjusted for other trace elements. The mechanism is needed to be further explored.

Information about the relationship between nickel concentrations and schizophrenia risk is limited. A nickel deficiency has been associated with type 1 and type 2 diabetes [36], and studies in the United States and Japan showed that diabetes mellitus was more common among patients with schizophrenia compared with the general population [37], which indicated that the nickel deficiency might be linked to schizophrenia.

Several limitations in the present study should be considered. First, our data presented changes of trace element levels in peripheral blood (serum), and further researches still need to adequately determine whether such changes reflect relevant alterations in the brain. Second, owing to the absence of some variables, including body mass index (BMI), smoking, dietary intakes and use of antipsychotics, potential confounding biases could not be fully adjusted and avoided. However, some study did not find any association between antipsychotic doses, duration of illness, and smoking status, and the changes of several trace elements, including manganese, selenium, iron, copper and zinc [17]. In addition, even if the socioeconomic and nutritional status of cases and controls are similar, it is still impossible to rule out the possibility that various genetic profiles might play a role in the schizophrenia risk [38].

The present study also has some significant advantages. The first strength is the match on age, sex and residential areas of patients and healthy controls. Another one is the simultaneous determination of 10 trace elements under the same experimental conditions.

## Conclusions

The findings showed lower levels of selenium, copper and higher levels of manganese in the schizophrenia patients compared with controls, though this might be influenced by several factors, including BMI, dietary intakes and use of antipsychotics. In order to control the potential confounding biases, further studies should take the above factors into consideration.

## Supporting Information

S1 Data Data underlying the findings in the present study. (XLS)
